# Test of theory of foraging mode: Goldcrests, *Regulus regulus*, forage by high‐yield, energy‐expensive hovering flight when food is abundant but use low‐yield, low‐cost methods when food is scarce

**DOI:** 10.1002/ece3.8205

**Published:** 2021-12-02

**Authors:** Rolf Åke Norberg

**Affiliations:** ^1^ Department of Biological and Environmental Sciences – Zoology University of Gothenburg Gothenburg Sweden

## Abstract

1. Here, I describe foraging behavior of goldcrests, *Regulus regulus*, based on eight years of field observation in a coniferous forest dominated by Norway spruce *Picea abies* in southwestern Sweden. The aim was to test predictions from theory on the choice of optimal foraging modes in relation to food availability.
2. Mortality from early November to early March amounts to 70–86% among goldcrests in the resident population, suggesting they are food‐limited in winter. Food‐limitation manifests itself as a shortage of time for foraging. It promotes the use of foraging methods that minimize the daily foraging time by maximizing the rate of net energy gain. It increases both individual survival and competitiveness. Elimination of competitors by exploitation occurs when an individual is able to support itself, while food density in the habitat is reduced to levels at which others cannot.
3. Theory shows that when food is abundant, high‐efficiency energy‐expensive *search* and *capture* methods give shorter daily foraging times than low‐efficiency low‐cost methods, whereas the latter gives shorter daily foraging times at food shortages (Norberg 2021). Hovering flight is extremely expensive in energy but results in high foraging efficiency. Hover‐foraging should therefore be used when food is abundant.
4. In autumn, there were 85.3 arthropods per kilogram of branch mass, as opposed to 12.9 in spring. The numerical decline of arthropods, their fat metabolism, and size‐biased predation by birds reduced the spring density of food for goldcrests to less than 15.1% of the autumn density.
5. Hover‐foraging occurred 5.29 times per minute in autumn but only 0.23 times per minute in spring, which is 4.4% of the autumn frequency.
6. Foraging conditions are favorable at midsummer because of long days, high temperatures, and an abundance of arthropod prey. Parent birds that were feeding fledglings gathered food at a high rate and hovered 5.42 times per minute. But adults with no young to feed were not compelled to maximize the rate of net energy gain and only hover‐foraged 0.52 times per minute, which is 10% of that of providers.
7. These results are highly consistent from year to year and in qualitative agreement with theory.

## Graphical abstract

Goldcrests minimize daily foraging time by using high‐efficiency energy‐expensive hover‐foraging when food is abundant but low‐efficiency low‐cost methods at food shortages.

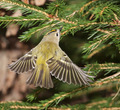

## 1 INTRODUCTION

The aim of this study is to describe foraging behavior of the goldcrest, *Regulus regulus* (L.), and to test predictions from theory on the choice of optimal foraging mode in relation to food availability (Norberg, 1977, 2021). Foraging consists of search for prey followed by pursuit, capture and handling, or capture for short. It is important to distinguish search from capture because an animal can search for all available prey simultaneously but captures them one at a time. Search time therefore increases with decreasing prey density, whereas capture time per item does not (Holling, 1965, 1968; MacArthur & Pianka, 1966; Schoener, 1971).

Norberg (1977) drew attention to the considerable amount of energy that animals expend on locomotion for foraging to cover their energy need and emphasized the accompanying increase in daily foraging time. Hunting on a decreasing prey population is a vicious cycle; the lower the prey density becomes, the more time and energy a predator must expend on foraging and the more food it needs.

Time for foraging may be a resource in limited supply (Holling, 1968). Animals usually have some repertoire of foraging techniques, so whenever the time required approaches the time available, time‐minimizing foraging methods should be used. It increases both individual survival and competitiveness. Elimination of competitors by exploitation occurs when an individual is able to support itself, while food density is reduced to levels at which others get short of time for foraging. S*urvival selection* favors individuals that minimize the daily foraging time or, equivalently, maximize the rate of net energy gain. When energy requirements are high, as during reproduction, time for foraging may be limiting even if food is abundant. So, *reproductive selection* may also favor minimization of foraging time.

In a theoretical model on the choice of optimal search modes, the optimization criterion is minimization of the daily foraging time or, equivalently, maximization of the rate of net energy gain. Different search methods were characterized exclusively by their food‐gathering efficiency and rate of energy expenditure. The higher the locomotor activity and associated rate of energy expenditure, the larger the space searched per time unit and the higher the encounter rate with food. A basic assumption therefore is that the higher the rate of energy expenditure of a *search* method, the more efficient it is. Results show that when food is abundant, high‐efficiency energy‐expensive *search* methods tend to give shorter foraging times than low‐efficiency low‐cost methods, whereas the latter does better at food shortages (Norberg, 1977).

That model has since been improved and extended to include the choice of optimal *capture* method as well. A fundamental premise is that the faster a capture method is, the more time it saves but the higher is its rate of energy expenditure. It turns out that food density in the habitat also determines which *capture* method gives the shortest daily foraging time. So, when food is abundant, high‐efficiency energy‐expensive *search* and *capture* methods give shorter daily foraging times than low‐efficiency low‐cost methods, whereas the latter gives shorter daily foraging times at low food densities (Norberg, 2021).

Here, I report on the foraging behavior of the goldcrest, *Regulus regulus*, based on an eight‐year fieldstudy in a forest of Norway spruce *Picea abies* (L.) H. Karst. in southwestern Sweden. Goldcrests are genuine insectivores all year round. They do not cache food in autumn for consumption in winter but have to subsist on the contemporary supply of insects and spiders and their eggs, larvae, and pupae. Arthropods do not reproduce in winter, so the amount of food for goldcrests decreases steadily from autumn to spring.

I have used published results from a comprehensive survey of the winter decline of arthropods in spruce canopies and conducted over six years near my goldcrest study site. Arthropod numbers declined by 85% from September‐October to March‐April (Jansson & von Brömssen, 1981). Based on the foraging theory (Norberg, 1977, 2021), I predict that goldcrests use high‐efficiency energy‐expansive foraging methods in autumn, when food is abundant, but not in spring when food is scarce.

Goldcrests hover both for search and capture of prey. Hover‐foraging is extremely expensive in energy but very efficient, which makes it a high‐yield energy‐expansive foraging mode. To test predictions from my 1977 and 2021 theories, I recorded the frequency of hover‐foraging in autumn, when food was abundant, and in early spring, when food was scarce. The prediction from theory is that the number of hovering flights per unit of time is high when food is abundant but not when food is scarce. When the frequency of hover‐foraging is high, the frequency of alternative foraging methods is reduced correspondingly. But I did not record the frequency of alternative methods.

I have no data on arthropod density in summer. But most passerine birds time their breeding so that they raise young when food is abundant, and most young fledge here just before midsummer. In addition, insects are very common at this time of year. So, from circumstantial evidence, I conclude that the density of arthropods in spruce trees is considerably higher at mid‐summer than during the February‐March observation period and probably matches, or even surpasses, the September‐October density. Considering also the many hours of daylight at mid‐summer, birds are not generally time‐limited and need not use strenuous, time‐minimizing foraging methods. But parent birds that are feeding young may be time‐limited and inclined to use high‐yield high‐cost foraging methods to maximize the rate of net energy gain and reproductive output. Therefore, I also recorded hover frequency at mid‐summer, distinguishing between parent birds that were feeding fledged young and adults that were not feeding any young.

## 2 MATERIAL AND METHODS

### 2.1 The goldcrest and focal foraging method

The goldcrest is the smallest bird in the Palearctic, with a mass of 5.9 g (mean of 63 birds weighed in August‐November in southwestern Sweden). The resting metabolic rate of birds scales with body mass to the power of 0.67 (Bennett & Harvey, 1987). Because the goldcrest is so small, it has a high mass‐specific metabolic rate and requires a high intake rate of food. In winter, it must eat 6–7 g of arthropods per day, which is more than its body mass (Thaler, 1973). Therefore, goldcrests must forage continuously during daylight hours in winter, which favors the use of time‐minimizing foraging modes.

The goldcrest is tightly bound to coniferous forests dominated by mature Norway spruce. Even though it is a genuine insectivore, about half of the population is resident throughout winter in Scandinavia, where subzero temperatures and a snow cover may last for months. At latitude 60°N in southern Finland, about 50% of the population migrated, most of them being juveniles. Among those that stayed, winter mortality from early November to early March averaged 70% across six winters (Hildén, 1982, p. 112). At latitude 60°N in southern Norway, mortality from early November to the end of March varied between 76% and 96%, with an average of 86% over six winters (Hogstad, 1984). The high mortality indicates that food is a limiting factor for winter residents.

Goldcrests are capable of hovering in still air—something few birds can and routinely do, and they are known to use it for foraging (Leisler & Thaler, 1982; Palmgren, 1932, p. 76; Thaler, 1973). Hovering flight is one of the most power‐demanding locomotion modes and expends energy at rates 10 times the basal metabolic rate (Pennycuick, 2008). But hover‐foraging is very effective. It is a well‐defined activity and easy to observe and recognize. Therefore, hover‐foraging is chosen as the focal foraging method.

### 2.2 Study site

The goldcrest study area is part of an indigenous forest of Norway spruce *Picea abies*, located near the small village of Bohult, 45 km east of Gothenburg at latitude 57.8°N in southwestern Sweden. The forest is rather open and consists of 15‐ to 20‐m‐tall trees. Except for spruce, there are occasional pine trees *Pinus silvestris*, junipers *Juniperus communis*, birches *Betula* spp., rowans *Sorbus aucuparia*, and willows *Salix caprea*. The undergrowth consists mainly of European blueberries (*Vaccinium myrtillus*), bog bilberry (*Vaccinium uliginosum*), lingonberry (*Vaccinium vitis*‐*idaea*), and heather (*Calluna vulgaris*).

### 2.3 Field observations

Arthropods that hibernate in spruce trees do not reproduce in winter, so food for goldcrests decreases steadily from autumn to spring. The aim is to compare the frequency of hover‐foraging between autumn and spring. I recorded foraging behavior in October, November, and early December in five years and in February, March, April, and May in four years. I also recorded hover frequency in June and July in seven years under favorable summer conditions with long days, high ambient temperature, and an abundance of food.

The number of daylight hours is identical on days at equal distance in time before and after the winter solstice. And the autumn and spring observation periods are about symmetrically located in relation to the winter solstice, from October 9 to December 9 and from February 20 to April 15 (Tables 4 and 5). Therefore, the temporal variation of day‐length is similar in the autumn and spring observation periods, albeit in a reversed order.

Ambient temperature tends to be lower in early spring than in late autumn. To eliminate potential effects of low temperature on foraging behavior, I recorded hover frequency only when temperature was above freezing—mostly between 0° and +5°C—both in autumn and spring.

Goldcrests might use different foraging behavior in different tree species and alter the allocation of time among them over winter. Therefore, I made observations of foraging in spruce only, which was the dominant tree.

After arriving at the study area, I detected goldcrests within 15 min or so and then could often maintain contact as long as desired. In autumn, winter and spring they often occur in mixed‐species foraging flocks together with crested tits (*Parus cristatus*), willow tits (*Parus montanus*), and coal tits (*Parus ater*). Their vocalization is louder than that of the goldcrest and facilitated detection.

When following goldcrests through their home‐range, I walked on foot but used skies when there was a snow cover—occasionally 70 cm deep. Under snowy conditions, foraging was recorded only when trees were completely free from snow due to a previous thaw or storm.

All observations of goldcrest foraging behavior were made on unmarked, wild birds. Observation distances were mostly 2–15 m. Except for close‐range observations, I used a 10× magnification Leica binocular. Goldcrests showed no sign of being disturbed but sometimes foraged within a meter of me. When I got an individual bird in plain view, I started talking observations into a tape recorder. I kept track of one bird at a time and recorded hover events but did not attempt to determine whether it hovered for search or for capture. When I lost view of the bird, if only for a short moment, I terminated recording. So a recording session always represents continuous observation of one individual. But I often shifted focal bird from one session to another, prioritizing visibility. In spring and summer, goldcrests sometimes sang while foraging, but I continued recording as long as they kept on foraging.

To be counted as hovering flight, there must be a distinct moment of standstill in mid‐air, however short. When a bout of hovers occurred in close succession, I counted each stop as a separate hover. Hovering makes a bird conspicuous, so I often detected it because it hovered. I did not count the initial hover but started recording immediately after it, not to overestimate hover frequency. When analyzing the tapes, I counted hover events and timed the recording session with a stopwatch.

Goldcrests shifted position frequently and often moved fast in spruce canopies, which are compact with poor visibility. Therefore, they were difficult to follow and many recording sessions were brief. I made a total of 1163 recording sessions, distributed among years and months, as shown in Tables 4, 5, 6. The total observation time was 12 h and 23 min. Session length varied from 2 s to 12.15 min and was 38 s on average.

## 3 RESULTS

### 3.1 Goldcrest diet

To enable testing of the theory of optimal foraging modes, I have collected background information from the literature regarding goldcrest diet, taxonomic composition of the arthropod fauna in spruce, and winter decrease in the arthropod number in spruce. In the following review of goldcrest diet, I emphasize studies made in Scandinavia at similar latitudes to that of my goldcrest study site at 57.8°N in southwestern Sweden. Diets have been analyzed in Finland and Norway, but not in Sweden.

In Finland, prey animals were identified from stomach contents of 42 goldcrests. They were collected in April, July, August, September, October, and November over a three‐year period in a spruce forest on the Finnish island of Åland at 60°N in the Baltic Sea (Palmgren, 1932). In all, 756 prey items were identified. Their taxonomic composition is listed here roughly in order of descending numbers: spiders, mostly *Philodromus aureolus*, *Clubiona* spp., *Xysticus* spp., *Pityohyphantes phrygianus* and Opiliones; Homoptera, mostly Aphididae and Psyllida; Diptera, mostly Chironomidae and Tipulidae; Psocoptera; Coleopteera, mostly Curculionidae; and cocoons and eggs of spiders and insects, most of which were hymenopterans and lepidopterans, with moths of the family Pyralidae predominating (Palmgren, 1932).

This study also examined the arthropod fauna on spruce branches collected in June, July, and August in the same three‐year period and forest. The goldcrest diet was similar to the taxonomic composition of the spruce fauna, except that some species of spiders, Psocoptera, Formicidae, and Brachycera occurred at lower proportions in the diet than in the natural population, whereas Opiliones, Coleoptera, Tipulidae, and Nematocera were overrepresented in the diet. Part of this discrepancy was attributed to differences in vigilance and escape ability between the different prey types (Palmgren, 1932, p. 73).

In Norway, goldcrest diet was determined from gizzard contents of 21 birds collected during a five‐month period from October through February in a spruce forest near Oslo at 60°N in southern Norway (Hogstad, 1984). Among 187 identified prey items in the pooled diet sample from all five months, spiders predominated and made up 60% by number. The remaining part, listed in order of descending numbers, consisted of Hemiptera 19.3%, Diptera 5.9%, Hymenoptera (parasitic) 3.7%, Psocoptera 3.2%, Coleoptera 3.2%, and Lepidoptera larvae 0.5%. Spiders made up an increasing proportion of the diet as winter progressed—from 45% in October to 78% in the combined sample from January and February (calculated from Table 2 in Hogstad, 1984). According to direct observations of foraging birds at close range in Austria, the winter diet of Goldcrests contains large numbers of springtails, Collembola (Thaler, 1973).

### 3.2 Taxonomic composition of arthropods in spruce canopies in southwestern Sweden

Here, I review published data on the taxonomic composition of arthropods in samples from spruce canopies in a forest located 25 km from the goldcrest study site and 40 km east of Gothenburg at 57.6°N in southwestern Sweden. The purpose is to examine whether the samples contain the prey species actually eaten by goldcrests.

Arthropod samples were collected between September 10 and October 10 and again between February 11 and March 7 in six consecutive winters, from autumn 1972 through spring 1978 (Jansson & von Brömssen, 1981). The arthropod sampling period overlapped in time with my goldcrest study in spring 1976, autumn 1976, and spring 1977.

Table 1 shows the occurrence of the various arthropod taxa in terms of number of animals per kilogram of needle‐carrying parts of spruce branches. The first two columns show the arithmetic mean density for each taxon, taken across the six autumn and the six spring samples, respectively. The two rightmost columns show the percentage composition by taxon before and after winter, calculated from the six‐winter average densities (based on Appendix 2 in Jansson & von Brömssen, 1981).

**TABLE 1:** Arthropod fauna on branches of spruce *Picea abies* in southwestern Sweden

Taxon	Number of animals/kg branch mass averaged across six winters		Average decline in per cent over 150 winter days	Percentage composition by taxon in samples from six winters	
	Before winter	After winter	%	Before winter %	After winter %
Opiliones	0.4	0.00	100	0.5	0.0
Araneidea	33.3	11.2	66.4	39.0	86.8
Psocoptera	38.2	0.15	99.6	44.8	1.2
Aphidoidea	6.1	0.06	99.0	7.2	0.5
Lepidoptera larvae	2.9	0.27	90.7	3.4	2.1
Coleoptera	1.7	0.06	96.5	2.0	0.5
Other insects	2.7	1.16	72.3	3.1	8.9
All taxa: Mean	85.3	12.9	84.9	100	100
*n*	6	6	–	–	–
*SD*, *SEM*	49.1, 20.0	5.0, 2.0	–	–	–

Note

Taxonomic composition and numerical decline through winter based on samples collected in September‐October and in February‐March over six consecutive winters.

The number of animals per kilogram of spruce branches before and after winter is the arithmetic mean number calculated for each taxon across all samples collected before, respectively, after each of six consecutive winters, from autumn 1972 through spring 1978. The average decline of the various taxa over five winter months was calculated from the six‐year average autumn and spring numbers, respectively, and expressed in per cent of the six‐year average autumn value for the respective taxon. The percentage composition by taxon refers to average numbers from all years. Table based on Appendix 2 in Jansson and von Brömssen (1981).

The taxonomic composition of arthropods before and after winter was Araneida 39% vs 86.8%; Psocoptera (mostly genus *Caecilius*) 44.8% vs 1.2%; Aphidoidea (mostly *Chermes abietis* and *Adelges laricis*) 7.2% vs 0.5%; Lipidoptera larvae (mostly the moth *Grapholita tedella*) 3.4% vs 2.1%; Coleoptera 2.0% vs 0.5%; Hymenoptera 1.1% vs 1.3%; Diptera larvae 0.6% vs 0.9%; and Psylloidea 0.3% vs 3.7% (based on Appendix 2 in Jansson & von Brömssen, 1981). Collembola were very numerous on spruce branches but because of their small size were not quantified (Jansson & von Brömssen, 1981, p. 84).

Here follows a review of an analyses of the taxonomic identity that I made of spiders in two of the aforementioned samples collected 22–23 September 1975 and 1–2 March 1976, containing 473 respectively 103 spiders (Norberg, 1978). *Philodromus aureolus* dominated and made up 39.1% vs 35.0% by number in the autumn and spring samples, followed by Linyphinae 14.8% vs 16.5%; Erigoninae 12.3% vs 10.7%; *Araneus sturmi* 7.8% vs 6.8%; *Clubiona* spp. 7.6% vs 2.9%; *Dictyna* sp. 4.4% vs 9.7%; *Xysticus* spp. 4.9% vs 0%; *Araneus cucurbitinus* 1.3% vs 2.9%; and *Diaea dorsata* 1.1% vs 1.0% (Table 2). The taxonomic composition of the natural spider population in spruce canopies remained fairly stable throughout the winter.

**TABLE 2:** Taxonomic composition of spiders on branches of spruce *Picea abies* in Southwestern Sweden, based on a before‐winter sample collected 22 and 23 September 1975 and an after‐winter sample from 1 and 2 March 1976

Family	Genus and species	Percentage	Percentage
		22–23 Sep	1–2 March
Dictynidae	*Dictyna* C. J. Sundevall sp.	4.4	9.7
Clubionidae	*Clubiona* P. A. Leatreille sp.	7.6	2.9
Thomisidae	*Diaea dorsata* (Fabricius)	1.1	1.0
”	*Xysticus* C. L. Koch sp.	4.9	0.0
”	*Philodromus aureolus* (Clerck)	39.1	35.0
Araneidae	*Araneus sturmi* (Hahn)	7.8	6.8
”	*Araneus cucurbitinus* Clerck	1.3	2.9
”	*Araneus* Clerck sp.	1.5	4.9
Linyphiidae	Subfam. Erigoninae	12.3	10.7
”	Subfam. Linyphiinae	14.8	16.5
Other	–	5.2	9.6
Total number of spiders		473	103

Note

Except for one individual spider that was 7 mm long, all spiders were 1.6–5 mm in body length. The size class 1.6–2.0 mm body length was commonest and made up 40% by number in the autumn sample and 42% in the spring sample. Based on Table 1 in Norberg (1978).

Because of a disproportionate decline of insects in winter (Table 1), the six winter average proportion of spiders in the arthropod samples from spruce in southwestern Sweden increased from 39% in September to 86.8% in March (Jansson & von Brömssen, 1981, Appendix 2). This change was reflected in the goldcrest diet in southern Norway, where the dietary proportion of spiders increased from 45% in October to 78% in January‐February (Hogstad, 1984).

Spruce branches sampled from November through March near Oslo at 60°N in southern Norway contained arthropods with species composition similar to that in southwestern Sweden (Hågvar & Hågvar, 1975). In southern Norway, *P*. *aureoles* made up 32% of the spider population on spruce branches in November and 29% in March (calculated from Table 3 in Hågvar & Hågvar, 1975). Similar proportions were found on spruce branches in southwestern Sweden, where *P*. *aureoles* made up 39.1% of the number of spiders in September and 35.0% in March (Table 2) (Norberg, 1978). In Finland, *Philodromus aureolus* was the most common spider both in spruce canopies and in the goldcrest diet (Palmgren, 1932).

The aforementioned data show that the arthropod fauna in spruce canopies in southwestern Sweden is very similar to those in Finland and Norway, where goldcrest diet was analyzed. And the goldcrest diet closely reflected the natural arthropod fauna and followed its seasonal fluctuations. Therefore, the arthropod samples from southwestern Sweden are reliable estimates of the amount of food available to goldcrests.

### 3.3 Decrease of arthropod density in spruce canopies over winter

In September‐October, spiders made up 60.4% and insects 39.6% of the number of arthropods in samples from spruce canopies in southwestern Sweden (means of pre‐winter samples from six winters, Table 1). But by February‐March, insect density had declined dramatically. The average decrease was 99.6% for Psocoptera, 99.0% for Aphidoidea, and 90.7 for lepidoptera larvae (means of post‐winter samples from six winters, Table 1) (from Appendix 2 in Jansson & von Brömssen, 1981).

As noted before, the large winter decline in insect number increased the six winter average proportion of spiders from 39% in September to 86.8% in March (Table 1) (based on Appendix 2 in Jansson & von Brömssen, 1981). But the absolute density of spiders declined by 72, 70, 57, 54, 71, and 70% in the six winters, and the average decline from September to March was 66%.

The population density of arthropods on spruce branches underwent a considerable and statistically significant reduction in each of the six winters (*p *< .001, Mann–Whitney *U*‐test; Jansson & von Brömssen, 1981). In Table 1, the six winter average densities of all taxa have been added together, yielding 85.3 arthropods per kg of needle‐carrying branch parts in autumn and 12.9 in spring, a reduction by 85% in about 140 winter days (Table 1; Figure 7). This decline was compared with goldcrest hover frequency (Tables 4, 5, 6).

To determine the effect of bird predation on winter mortality of arthropods, a field experiment was run through one winter in the forest, where arthropods were sampled (Askenmo et al., 1977). Randomly selected spruce branches were enclosed with nets to prevent birds from foraging on them from mid‐October to the beginning of March. In March, spider density had declined by 57% on unprotected control branches but only by 34% on the protected ones. The difference indicates that birds consumed 23% of the spiders present in the previous autumn (Askenmo et al., 1977). Birds preyed selectively with respect to prey size and took spiders with a combined cephalothorax–abdomen length over 2 mm more often than smaller spiders (Askenmo et al., 1977; Jansson & von Brömssen, 1981). Therefore, the average spider was smaller in spring than in autumn. But the taxonomic composition of spiders on non‐experimental spruce branches was rather stable throughout winter (Norberg, 1978). So birds exerted no selection with regard to spider taxonomy.

Two of the aforementioned arthropod samples from spruce canopies in southwestern Sweden were used to measure the decrease in the energy content of spiders in winter, using micro‐bomb calorimetry. Over 126 winter days, from 10 October 1972 to 12 February in the following spring, the energy content per unit of body mass decreased by 13% due to metabolism of body fat (Norberg, 1978). The reduced energy density and the smaller average body size of spiders after winter worsen the goldcrest's food situation in early spring—over and above the effect of reduced arthropod number.

### 3.4 Goldcrest foraging behavior

#### 3.4.1 Movement in winter territory

In the non‐breeding season, goldcrests often occurred in mixed‐species foraging flocks together with *Parus montanus*, *P*. *cristatus*, *P*. *ater*, and *Certhia familiaris*. The group association was loose, however, and goldcrests also formed flocks by themselves, containing two to eight birds, similar to their winter behavior in Finland (Hildén, 1982) and Norway (Hogstad, 1970, 1984). Sometimes, single birds foraged alone. The daily movement of a goldcrest flock was mostly confined within a polygon 200–300 m across, like in Norway (Hogstad, 1970, 1984) and Austria (Thaler, 1973).

The previously study by Hogstad (1970) in Norway found that the habitat area covered daily by flocks of goldcrests decreased from 5–6 ha in November‐December to 1.5–3 ha in January. And goldcrests reduced their speed of movement from 300 m/h in November‐December to 150 m/h in January (Hogstad, 1970). This is a gradual shift from efficient foraging behavior that covers a large habitat space per unit time with the use of high travel speeds at high rates of energy expenditure in autumn, when food is abundant, to covering a smaller habitat space per unit time at reduced travel speeds and lower rates of energy expenditure in spring when food is scarce. It is in line with predictions from foraging theory (Norberg, 1977, 2021).

#### 3.4.2 Locomotion modes during foraging

Goldcrests foraged uninterruptedly the entire day in late autumn, winter, and early spring and continued well into dusk after it had become too dark for me to observe their activities. I rarely saw them sitting still, resting, or preening. In summer, birds that were feeding newly fledged young also foraged intensively. But adults that were not feeding young often rested, preened, and sang. This indicates that foraging was time‐limited in winter but also in summer for parents that were feeding fledglings.

Foraging goldcrests mostly frequented the needle‐carrying parts of spruce branches. They moved upward in trees more often than downward, using small hops, wing‐assisted hops, and short flights. This habit has been noted also in Norway (Haftorn, 1986). It is consistent with a theory on minimization of energy expenditure during locomotion among trees. The point is that potential energy gained while climbing upward in a tree can be recovered and converted into distance flown, provided that the flight path is sloping downward, which reduces flight muscle power output (Norberg, 1981, 1983). For this locomotion mode to be cheaper in energy than alternative modes, the upward movement must occur predominantly by non‐flight locomotion, such as hopping and climbing. An additional benefit of moving upward in trees is that it facilitates detection of prey on the underside of branches and twigs where arthropods often reside.

**FIGURE 1:**
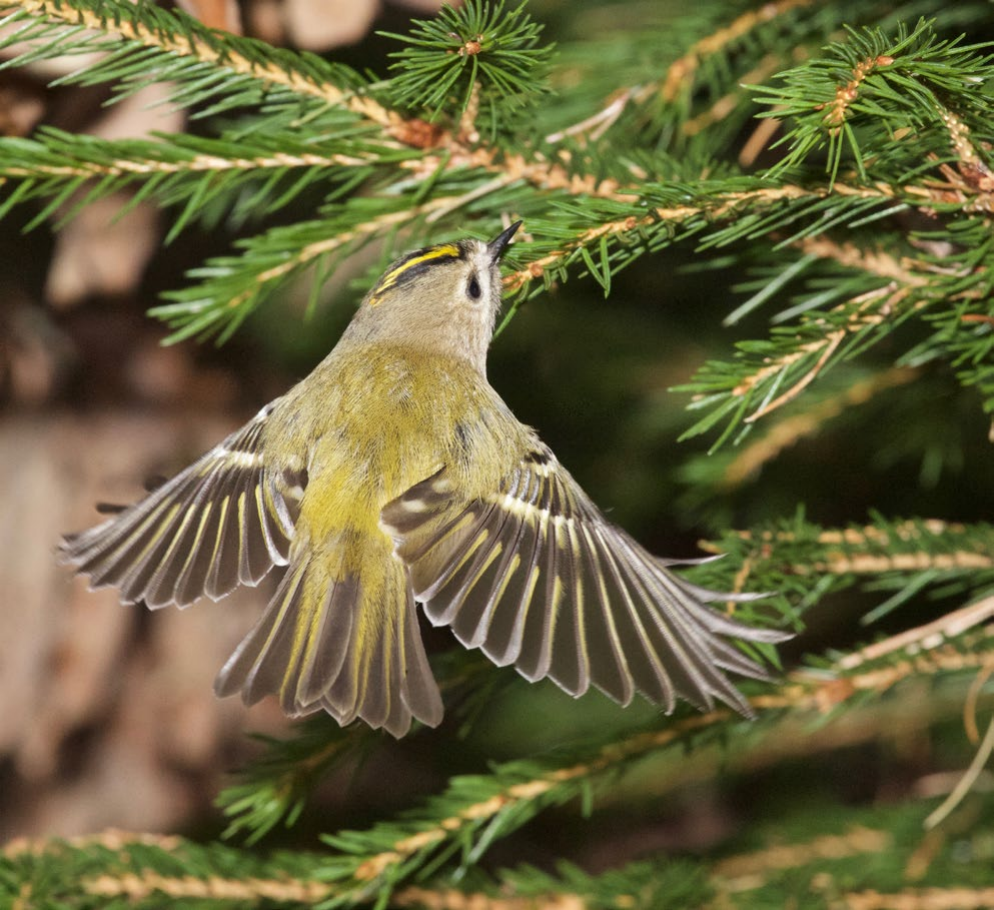
Goldcrest hover‐searching in front of a branch of spruce *Picea abies*. Nov 8, 2013. Photo: R. Åke Norberg

**FIGURE 2:**
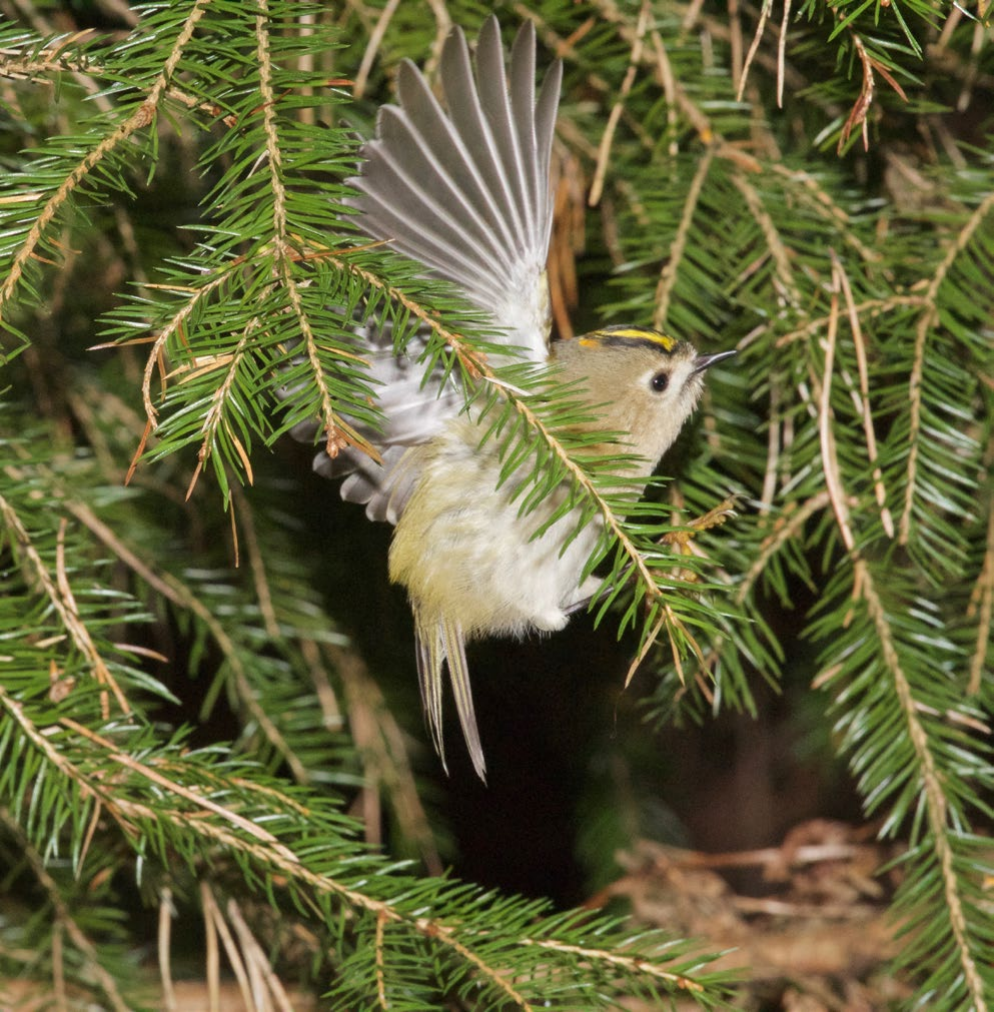
Hover‐search in a narrow space amidst twigs and needles inside the canopy of spruce. Nov 22, 2013. Photo: R. Åke Norberg

**FIGURE 3:**
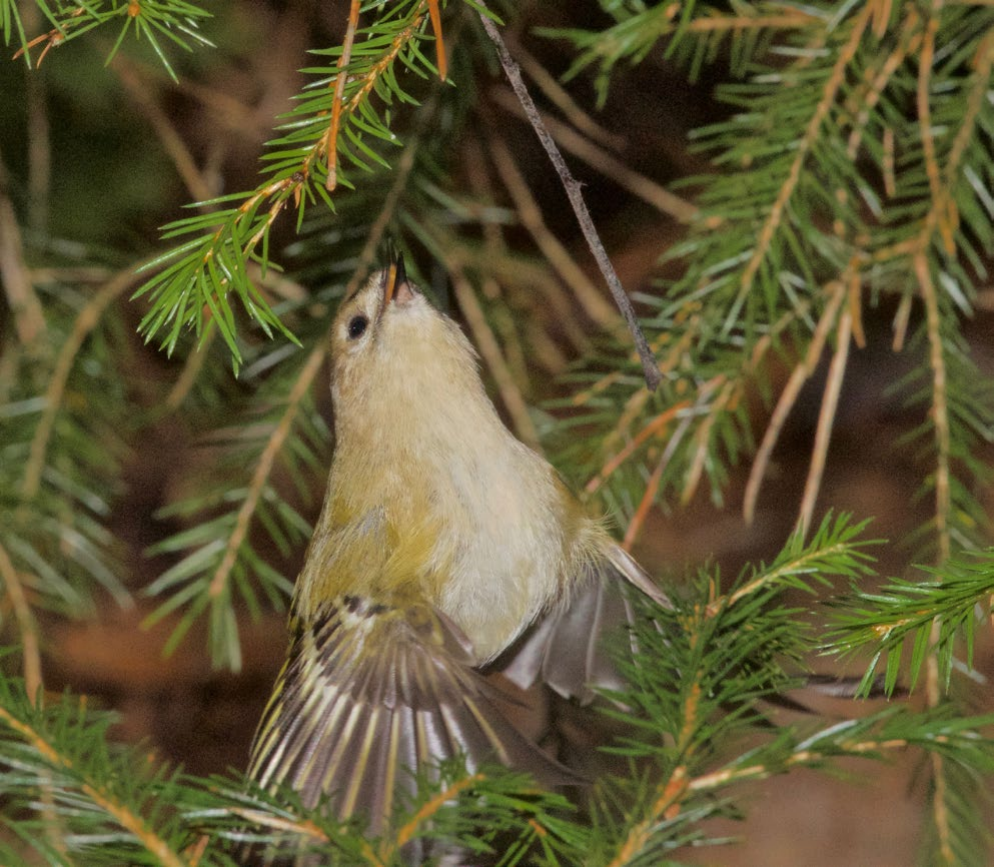
Hover‐capture. Goldcrest in hover about to pick a prey off a spruce twig. Nov 22, 2013. Photo: R. Åke Norberg

**FIGURE 4:**
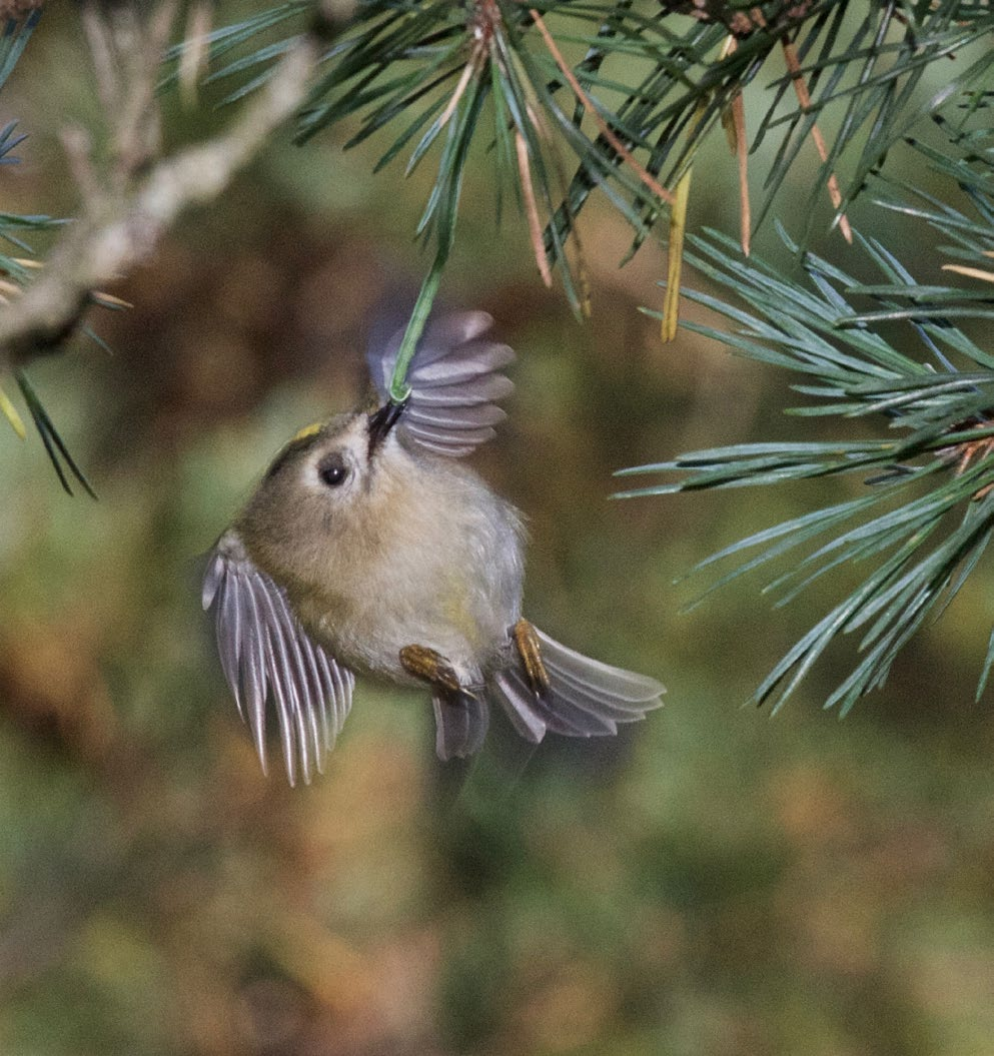
Hover‐capture. Goldcrest in hover, taking a pine moth caterpillar (*Bupalus piniarius*) from needles of pine *Pinus silvestris*. Oct 14, 2015. Photo: R. Åke Norberg

**FIGURE 5:**
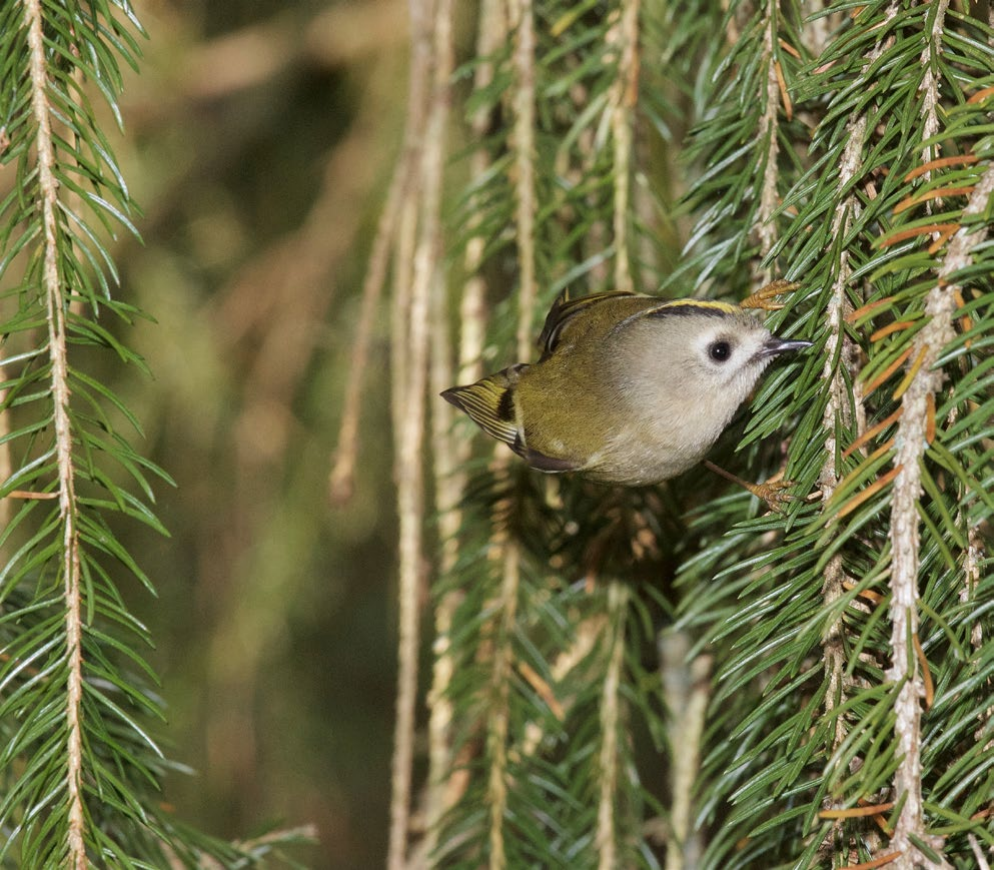
Foraging by hopping and clinging requires less energy than hovering but is less efficient. Oct 14, 2015. Photo: R. Åke Norberg

Goldcrests moved with great agility and used a mixture of locomotion modes and feeding postures such as hopping on top of branches, clinging to the sides of branches and twigs (Figure 5), and hanging upside‐down underneath twigs. During these movements, they were continuously picking up prey items at a high rate. They also searched for and captured prey by hovering flight. Goldcrests always picked prey off the substrate (Figures 1, 2, 3, 4, 5), and I never saw them capture any flying insect. Winter crane flies Trichoceridae often swarmed in mild weather, but I never saw goldcrests attempting to catch any in flight. On two occasions though, I saw a goldcrest fly out from a tree and inspect a spinning, winged spruce seed in slow autorotational descent (Norberg, 1973), but without seizing it.

#### 3.4.3 Locomotor activity of foraging goldcrests

During one observation session in November and one in March, I recorded every movement that resulted in a change of position, regardless of distance moved. I counted each hop in a series of hops, each flight within a tree, and each flight between hover–stops in a bout of hovering flights. A recording session includes movements within a tree but not flights between trees. On average, goldcrests made 54 movements per minute (Table 3).

**TABLE 3:** Locomotor activity of foraging goldcrests

Date		Number of sessions	Number of moves	Obs time in minutes	Moves per minute
1977	November	10	319	6.42	49.69
1978	March	23	517	8.95	57.77
Total		33	836	15.37	54.39

Note

Every movement, including hops and flights, that resulted in a change of position was counted, regardless of distance moved. The average number of moves per minute at bottom right was calculated from the sums of the November and March numbers of moves and observation times, respectively.

#### 3.4.4 Hovering flight

Hovering flight is the most energy‐expensive locomotion mode that goldcrests use. It requires about 10 times more energy per unit time than the basal metabolism (Pennycuick, 2008) but generates high foraging yield, as explained below.

During hovering flight, the body is upright with the long body axis inclined 50–60° to the horizontal (Figures 1 and 2). In downstroke, the wingbeat plane is inclined ca. 30° to the horizontal and is about perpendicular to the long body axis, as in level flight. In the upstroke, the wings are strongly flexed and retracted close to the body. And the flight feathers are passively rotated in the nose‐up sense by the relative airflow, letting air through the wing. I counted the number of wing‐beats during a time interval of 1 s in video recordings of randomly selected individual goldcrests in hovering flight. The wing‐beat frequency was very constant across individuals and averaged 21 wing‐beats per second (*s* ^2^ = 0.5; *n* = 17).

Goldcrests use “asymmetric hover” in which the downstroke is the power stroke and the upstroke an aerodynamically inactive recovery stroke that brings the wings back to the starting position for the downstroke. Other passerine species that hover all use “asymmetric hover.”

Hummingbirds are different. They use “symmetric hover” or “normal hover” where downstroke and upstroke are kinematically symmetric with the wingtip tracing out a horizontal figure of eight. During the morphological upstroke, their wings are fully extended and inverted, dorsal side facing down, and contribute 25% of the upward force generated in a full wingbeat cycle in the rufous hummingbird *Selaphorus rufus* (Warrick et al., 2005), 35% in Anna's hummingbird *Calypte anna* (Wolf et al., 2013).

#### 3.4.5 Hover‐search and hover‐capture

The following characterization of hover‐foraging is based on close‐range observations through the optical viewfinder of a Canon EOS 200D APS–C camera with an EF 70–200 mm, f/4 L, IS II USM lens while taking still photographs at 2–4 m distances in autumn 2013–2018. In addition, I made frame‐by‐frame analyses of video clips of hover‐foraging goldcrests recorded at 50 frames/s.

A hover event may be a single standstill in mid‐air in front of a twig, after which the goldcrest closes in on a detected prey to pick it up while still hovering or else breaks off into normal flight. So hovering flight is used both for search of prey and for capture, and a hover‐search is often, followed by hover‐capture. This happens very fast, so in most cases, I could not distinguish between hover‐search and hover‐capture. Therefore, I just recorded instances of hovering flights.

A single hovering event typically lasts 1–3 s. But goldcrests often search for prey by making a series of three to five hovers separated by short flights. It usually advances sideways or downward 2–20 cm between halts, tracing the tree periphery at a distance of about 5 cm (Figures 1, 2, 3). Some old spruce trees have long and pliant pendulous twigs and goldcrests often descend along them in near‐hovering flight, interrupted by distinct hover stops. Hover‐search is particularly efficient because otherwise the bird would have to cling on to vertically hanging twigs while searching one spot at a time, which is time‐consuming (Figure 5). Hovering flight occurs also in remarkably narrow spaces among branches in the interior of spruce canopies (Figures 2 and 3).

**FIGURE 6:**
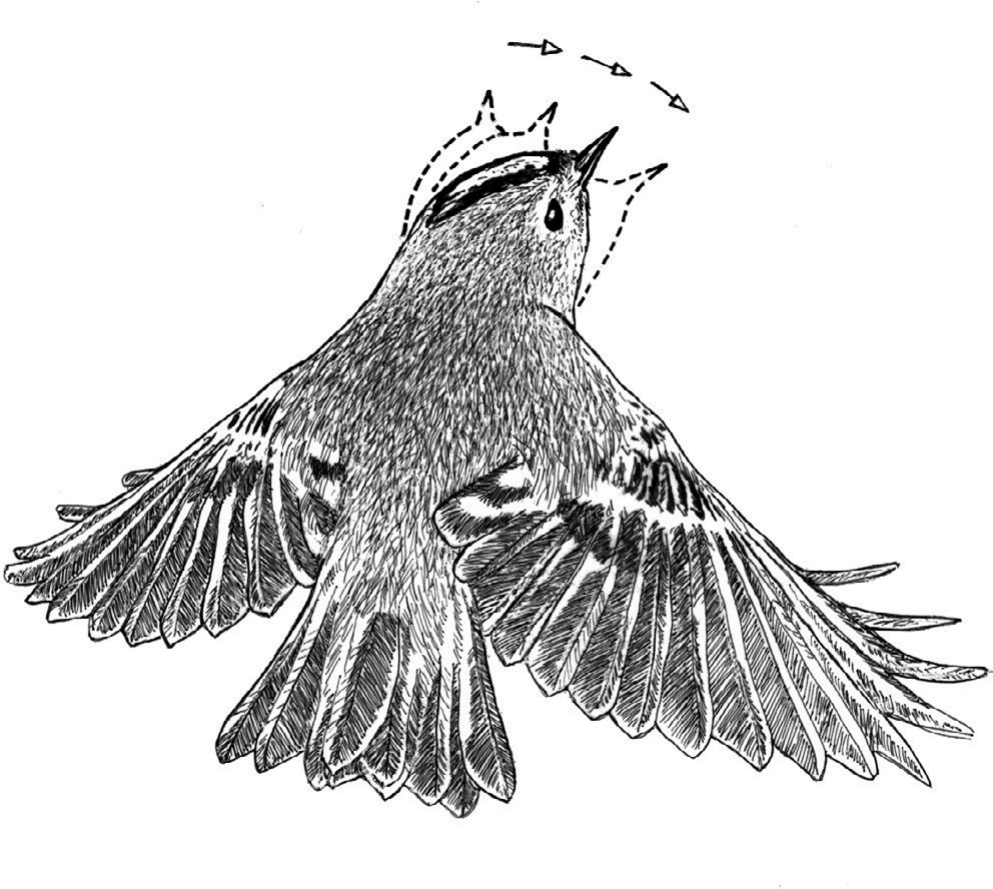
Goldcrest in hovering flight, scanning for prey. Based on Figure 1, observations and frame‐by‐frame analyses of video clips. During stationary hovering, goldcrests often scan for prey by turning the head sideways, shifting gaze in small angular steps, the so‐called saccadic head movements. A saccade typically consists of three to four quick head turns separated by brief but distinct stops for visual fixation, lasting on average 0.204 s. Keeping the head stationary, if only briefly, stabilizes the retinal image by eliminating self‐induced movements of optical images across the retina, which facilitates prey detection

When hovering, goldcrests often scan for prey by turning the head in small angular steps, the so‐called saccadic head movements. A saccade typically consists of three to four quick head turns separated by brief but distinct stops for visual fixation (Figure 6). Analysis of video recordings show that the time during which the head is kept still at a fixed heading angle between head turns is on average 0.204 s (*s* ^2^ = 0.0007; *n* = 14). Keeping the head stationary in a fixed heading angle, if only briefly, eliminates self‐induced movements of optical images across the retina. It ensures visual gaze stabilization and facilitates detection of prey.

When a goldcrest exits a tree, it often hovers in front of a branch before breaking off to fly to another tree. And when arriving to a tree it often hover‐searches at the canopy periphery before alighting. In fact, goldcrests often hover both when leaving a tree and when arriving at the next one. This is a time‐saving search technique.

### 3.5 Advantages with hover‐foraging

1. The needles of spruce twigs are directed obliquely outward and form 40–60° with the twig axis. When a bird sits on top of a branch, looking distally, the needles may conceal prey items underneath. Hovering provides an observation platform in mid‐air, which enables the bird to look in between the needles and search a larger space than otherwise possible (Figures 1 and 3).
2. Hovering provides a moment of standstill in mid‐air. It eliminates self‐induced movements of optical images across the retina and facilitates detection of prey.
3. Hovering saves time because a bird that hovers can move more quickly between foraging sites than one that alights on them (Figures 1, 2, 3, 4, 5) (Pyke, 1981).
4. Gleaning prey off vegetation in hovering flight increases capture success because it gives less time for prey to escape than if the bird alights first.

Goldcrests use hovering flight both for search and capture. Hover‐search expends more energy per unit of time than other search methods. But it is more efficient and enables the bird to search a larger space per unit of time than energy cheaper methods do. Likewise, hover‐capture expends more energy per unit of time than do other capture methods but give higher success rate and takes less time per capture event.

Theory shows that the advantage of using one method or the other depends on food availability. When food is abundant, foraging by hovering flight maximizes the rate of net energy gain and minimizes the daily foraging time. This is because the time saved by hover‐foraging can be used to procure additional food. And it is easier to find enough food to make up for the energy expended by hovering, and get a surplus, when food is abundant than when it is scarce. But when food is in short supply, low‐efficiency low‐cost methods are likely to give higher rates of net energy gain and shorter daily foraging times. This applies to search and capture methods alike (Norberg, 2021).

### 3.6 Hover frequency in relation to arthropod population density

Here, I relate the frequency of hover‐foraging to the density of arthropods in spruce. Tables 4, 5, 6 show hover frequency in autumn, spring, and summer.

**TABLE 4:** Hover frequency in late autumn

Date		Number of sessions	Number of hovers	Obs time minutes	Hovers/minute
1976	October 17–31th	325	1134	220.53	5.14
”	November 13th	36	125	18.30	6.83
1977	October 9th	26	31	12.18	2.55
”	November 5–26th	93	280	72.45	3.86
1978	November 18th	3	14	1.58	8.86
1979	October 13th	18	106	14.68	7.22
”	November 4–18th	29	108	13.93	7.75
”	December 9th	6	24	3.00	8.00
1980	October 26th	8	32	3.95	8.10
	November 5–23rd	18	93	7.80	11.92
Total		562	1947	368.40	5.29

Note

Horizontal rows list number of observation sessions, number of hovers, and observation time in a given month and year. The monthly hover frequency in the rightmost column was calculated from the total number of hovers and the entire observation time in the respective month and year. Bottom row shows sums across months and years. The overall average hover frequency at bottom right was calculated from the total number of hovers and the entire observation time added across months and years.

**TABLE 5:** Hover frequency in early spring

	Date		Number of sessions	Number hovers of	Obs time minutes	Hovers/minute
1976	February	26–28th	26	5	20.57	0.24
”	March	6–28th	27	7	23.67	0.30
”	April	7th	4	6	5.50	1.09
1977	February	20th	56	8	28.98	0.28
”	March	5–26th	159	17	117.43	0.14
”	April	15th	40	5	18.42	0.27
1978	February	26th	47	5	22.58	0.22
”	March	5th	28	5	12.40	0.40
1981	April	4–5th	39	6	29.33	0.20
Total			426	64	278.88	0.23

Note

Horizontal rows list number of observation sessions, number of hovers, and observation time in a given month and year. The monthly hover frequency in the rightmost column was calculated from the total number of hovers and the entire observation time in the respective month and year. The bottom row shows sums across months and years. The overall average hover frequency at bottom right was calculated from the total number of hovers and the entire observation time added across months and years.

**TABLE 6:** Hover frequency in summer

Date			Number of sessions	Number of hovers	Obs time minutes	Hovers/minute
Adult birds feeding fledglings						
1980	June 27–28th	45	116	23.40	4.96	Fed ≥7 fledglings
1981	June 24th	41	37	15.03	2.46	Fed ≥8 fledglings
1982	June 19th	5	13	3.00	4.33	Fed fledglings
1984	July 19th	24	166	19.88	8.35	Fed fledglings
Total	1980–1984	115	332	61.31	5.42	Fed fledglings
Adult birds with no young to feed						
1977	June 12th	19	8	6.43	1.24	Fed no young
1978	June 18th	4	0	3.92	0.00	Fed no young
1979	June 19–27th	34	6	19.02	0.32	Fed no young
”	July 1–2nd	3	3	3.63	0.83	Fed no young
Total	1977–1979	60	17	33.00	0.52	Fed no young

Note

Top half of table shows results for adult birds foraging busily and feeding a clutch of fledged young. Bottom half refers to adult birds foraging but with no young to feed. Horizontal rows list number of observation sessions, number of hovers, and observation time in a given month and year. The monthly hover frequency in the rightmost column was calculated from the total number of hovers and the entire observation time in the respective month and year. Bottom row shows sums across months and years. The overall average hover frequency at bottom right was calculated from the total number of hovers, and the entire observation time added across months and years.

#### 3.6.1 Autumn

**FIGURE 7:**
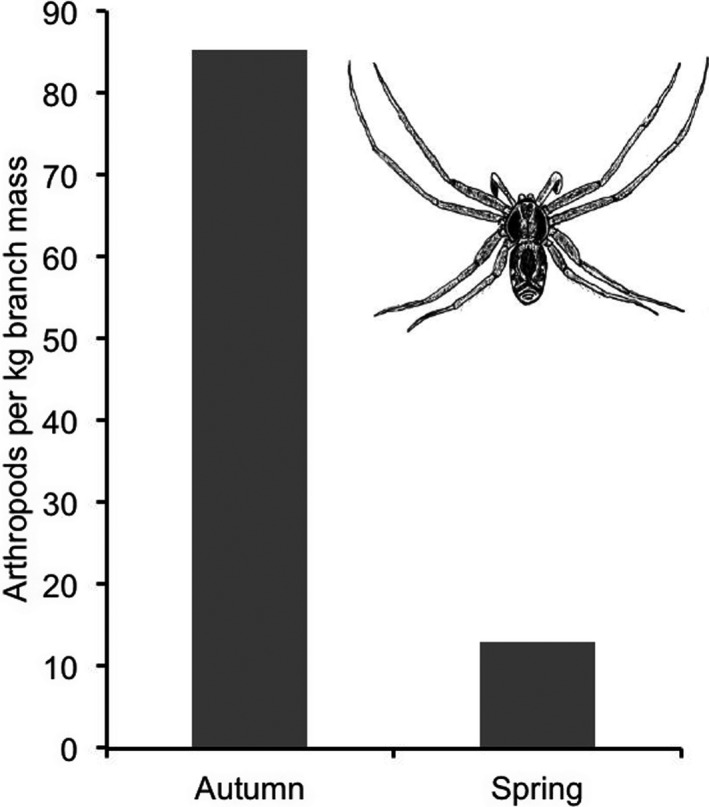
Number of arthropods per kilogram of needle‐carrying branch parts of spruce *P*. *abies* in autumn and spring, averaged across data from six winters (Table 1). Metabolism of body fat by spiders in winter reduced their energy content per unit of body mass by 13%. And spiders were smaller, on average, in spring than in autumn due to size‐biased predation by birds. Therefore, less food was available to goldcrests in spring than suggested by the mere number of arthropods. The inset shows *Philodromus aureolus*, the commonest spider in spruce canopies. The difference in arthropod number between autumn and spring is highly significant (*p* < .001, Mann–Whitney *U*‐test)

**FIGURE 8:**
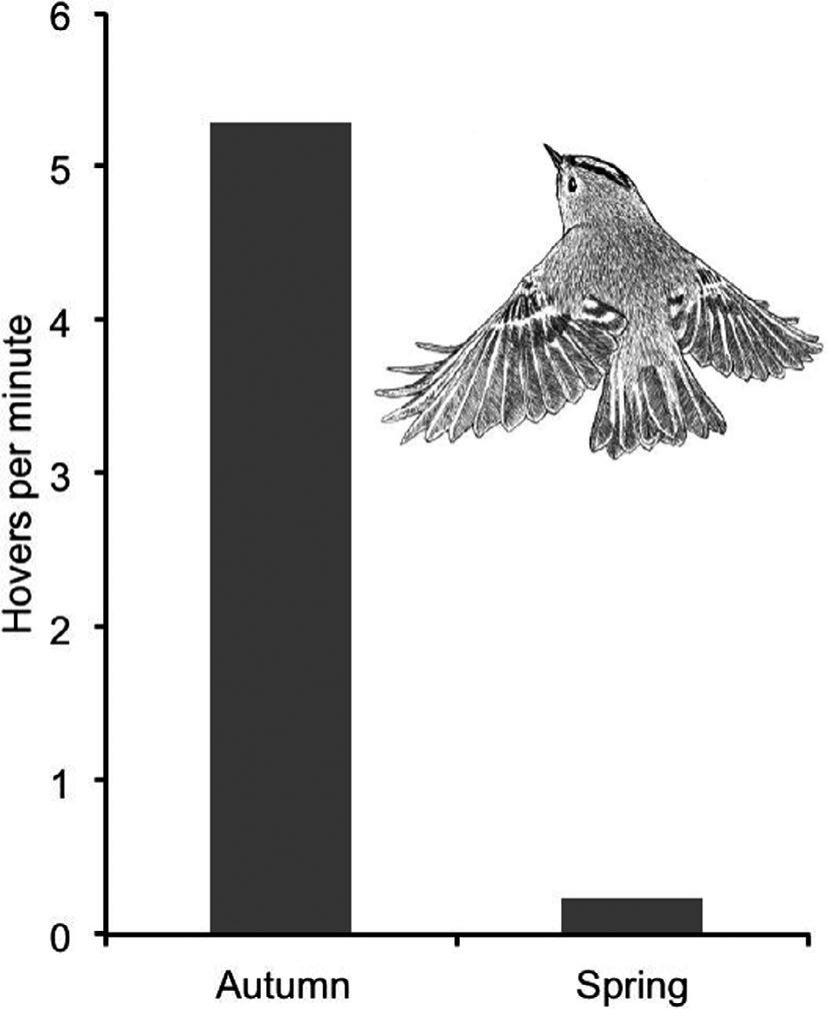
Goldcrest hover frequency in spruce in autumn and spring, averaged across data from five and four years, respectively (Tables 4 and 5). The difference in hover frequency between autumn and spring is highly significant (*p* < .00004, two‐tailed randomization test)

In September‐October, there were 85.3 arthropod animals per kilogram of branch mass, as averaged across autumn samples from six years (Table 1). Ten monthly hover frequencies, recorded in October, November, and December over five years, varied between 2.55 and 11.92 hovers per minute and gave a ten‐month average of 5.29 hovers per minute. It corresponds to 11 s between hovers (Table 4; Figures 7 and 8).

#### 3.6.2 Spring

In February‐March, there were 12.9 arthropod animals per kilogram of branch mass, as averaged across spring samples from six years (Table 1). It is 15.1% of the autumn value 85.3. But the spider metabolism of body fat reserves over winter reduced the energy content per unit of spider‐body mass by 13% (Norberg, 1978). And spiders were smaller, on average, in spring than in autumn due to size‐biased predation by birds, as detailed earlier. Therefore, food for goldcrests decreased more over winter than indicated by the mere numerical decline of arthropods, so the actual density of food in spring was less than 15% of the autumn value.

Nine monthly hover frequencies, recorded in February, March, and April over four years, varied between 0.14 and 1.09 hovers per minute and gave an average of 0.23 hovers per minute. It is 4.4% of the autumn frequency 5.29 and corresponds to 4 min and 18 s between hovers (Table 5; Figures 7 and 8). The difference in hover frequency between spring and autumn is highly significant (*p* < .00004, two‐tailed randomization test).

#### 3.6.3 Summer

Hover frequencies were recorded in late June and in July. There are no data on population density of arthropods for these months, but arthropods had most likely recovered numerically from the winter decline. This is also suggested by the fact that goldcrest nestlings fledge in late June, an event normally timed to occur when food is abundant.

**FIGURE 9:**
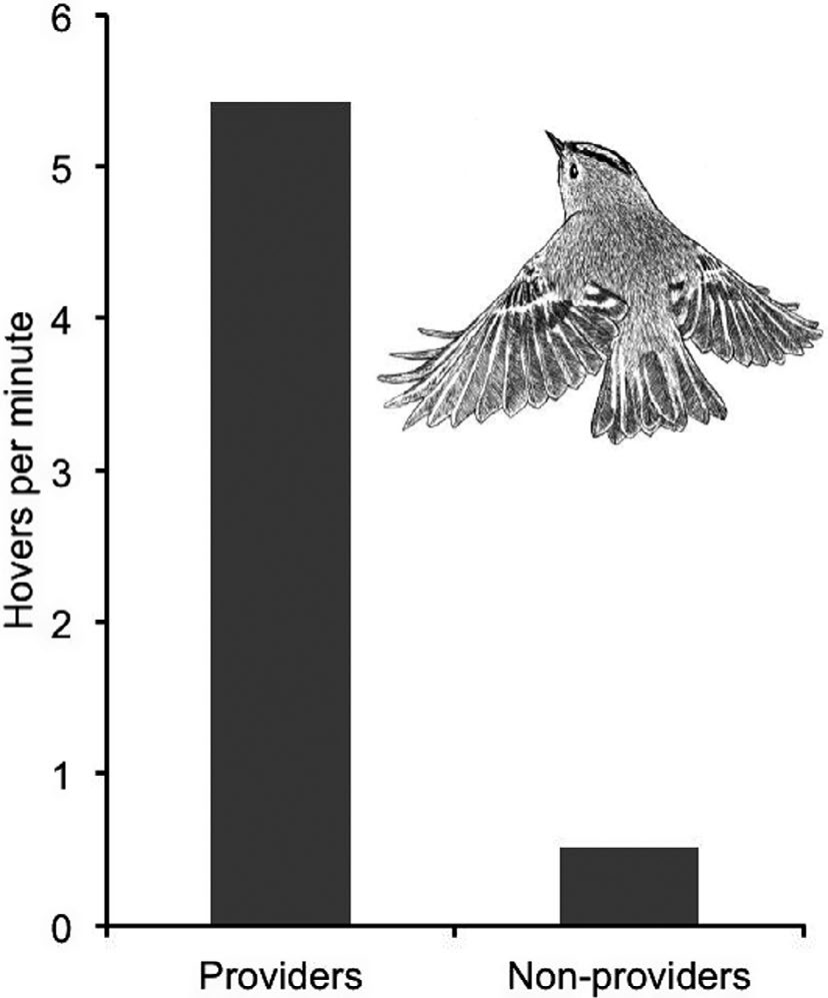
Hover frequency in spruce in summer by adult goldcrests that were feeding newly fledged young (providers) versus adults with no young to feed (non‐providers), averaged across four and three years, respectively (Table 6). All records were made in June and July in the same forest area. Providers and non‐providers faced identical environmental conditions, which were favorable because of high ambient temperature, long days, and an abundance of arthropods. The difference in hover frequency reflects different foraging efforts. Time was probably limiting to providers, promoting efficient but energy‐expensive hover‐foraging that minimizes foraging time when food is abundant. The difference in hover frequency between providers and non‐providers is statistically significant (*p* = .0286, two‐tailed randomization test)

I recorded foraging behavior of birds from each of two categories. One contained adult birds that were busily foraging and feeding a brood of newly fledged young at a high rate. The fledglings made no attempt to forage themselves but stayed tightly together and followed the parents closely, begging persistently for food. In one case, I saw at least 7 fledglings being fed and another brood contained at least 8 fledglings. It was difficult under field conditions to count fledglings that were constantly moving in dense spruce canopies. The numbers that I report are those that I could determine with certainty, and they most likely underestimate the real clutch sizes. The 19th July 1984 recording in Table 6 probably concerns a second brood. Four monthly hover frequencies, recorded in June and July in four years, varied between 2.46 and 8.35 hovers per minute and gave a four‐month average of 5.42 hovers per minute (Table 6; Figure 9). This is strikingly similar to the October–November frequency of 5.29 hovers per minute. Food demand obviously determined foraging effort. And because food was abundant in summer, hover‐foraging was favored by providers.

The other category was adult birds with no young to feed. They foraged at a leisurely pace and were never seen to visit any nest or feed any young. Four monthly hover frequencies recorded in June and July in three years varied between 0 and 1.24 hovers per minute and gave a four‐month average of 0.52 hovers per minute (Table 6; Figure 9). This is similar to the February–April frequency of 0.23 hovers per minute. The difference in hover frequency between providers and non‐providers is statistically significant (*p* = .0286, two‐tailed randomization test).

## 4 ALTERNATIVE EXPLANATIONS

According to the working hypothesis, seasonal differences in hover frequency depend on differences in food density. But there could be other causes. Here, I evaluate five alternative explanations.

### 4.1 Age‐related hovering propensity

The goldcrest is a partial migrant in Norway and Finland where about half of the population leaves in autumn. The vast majority of autumn migrants are hatch‐year birds and males outnumber females (Hildén, 1982; Lifjeld, 1982). The age and sex distribution among autumn migrants from Sweden are probably similar to those in Norway and Finland. Therefore, my study population most likely contained a higher proportion of hatch‐year birds in autumn than in spring. And if hatch‐year birds would hover more often than adults, it might explain the high hover frequency in autumn.

Migration from Sweden starts in mid‐September, peaks around mid‐October, and is completed by the end of October (Enquist & Pettersson, 1986 p. 151; Karlsson, 1992, p. 30, 41). The proportion of hatch‐year birds in the resident population is therefore lower in November and December than in October. But hover frequency was nevertheless consistently higher in November and December than in October in all comparisons between months (Table 4).

Arthropods were abundant in June and July, like in autumn. Goldcrests that were feeding fledglings hovered on average 5.42 times per minute, which is similar to the autumn frequency 5.29. But under identical environmental conditions, adult birds with no young to feed hovered 0.52 times per minute, which is similar to the spring frequency 0.23 (Tables 4, 5, 6). So, age and sex do not explain the seasonal variation in hover frequency.

### 4.2 Ambient temperatures and daytime lengths

A study near the city of Oulu at 65°N in northern Finland found that goldcrests hovered less frequently at low ambient temperatures in winter. It was suggested that the heat produced by flight muscles during hovering might not compensate for the convective heat loss to the hovering‐induced airflow past the body. Therefore, additional energy would be required for thermoregulation and make hovering flight more expensive in energy and less profitable at low ambient temperatures (Alatalo, 1982).

The study was carried out from October 1975 to March 1976, but most observations were made in December and January. There is no information on hovering frequency in relation to time in winter. But in the study area, near the city of Oulu, ambient temperature shows a declining trend from October through February. Food for goldcrests also decreases over winter. Therefore, low temperature and low food density are more likely to co‐occur late than early in winter. Because hover frequency decreases with decreasing food density (see before), the observed correlation between ambient temperature and hover frequency is not a cause‐and‐effect relationship, but rather arises because both temperature and food decrease through winter.

To avoid effects of differences in ambient temperature between the autumn and spring observation periods, I only recorded foraging behavior on days when temperatures were above freezing—mostly between 0° and +5°C both in autumn and spring. So, ambient temperature does not explain the temporal change in the frequency of hover‐foraging.

The autumn and spring observation periods are about symmetrically located before and after the winter solstice. Therefore, the temporal variation in day‐length is similar during the autumn and spring observation periods, albeit in a reversed order. Yet, hover frequency dropped from 5.29 in autumn to 0.23 in spring. And in summer, with long days and high ambient temperatures, goldcrests that were feeding young hovered 5.42 times per minute, while adults with no young to feed hovered 0.52 times per minute (Tables 4 and 6). So, day length did not determine hover frequency.

### 4.3 Composition of the arthropod fauna in spruce canopies

The arthropod fauna in spruce canopies changed considerably from September to March. The largest shift was a decrease in insect number by about 90%, whereas spiders fared better and declined by 66% (Table 1). The disproportionate reduction of insect density increased the proportion of spiders in the arthropod fauna from 39% before winter to 87% after winter. The taxonomic composition of spiders remained almost constant throughout winter and so did not affect the frequency of hover‐foraging (Table 2).

The scarcity of insects after winter might cause goldcrests to abandon hover‐foraging. But insects disappeared early and were almost absent by 30 November, whereas spiders declined gradually through winter (Jansson & von Brömssen, 1981; see their Figures 2 and 4 and Appendix 2 for the 30 November data). Goldcrests nevertheless hovered consistently more often in November and December than in October, despite the higher insect density in October (Table 4).

Insects were common at mid‐summer and then goldcrests that were feeding young hovered 5.42 times per minute, while adults with no young to feed hovered 0.52 times per minute (Table 6). As insects were equally available to them all, the composition of the arthropod fauna did not determine the frequency of hover‐foraging.

### 4.4 Prey depletion in hover‐accessible sites

Hover‐foraging might direct exploitation specifically to canopy parts that are particularly accessible by hovering flight. The high hover frequency in autumn could thus cause disproportionate depletion of prey in hover‐accessible sites and make them less rewarding. The reduced hover frequency in spring could thus be due to a shift to foraging sites that cannot be accessed by hovering flight. But the depletion rate of prey in the outer, exposed, and hover‐accessible sites is slowed down in winter because those are the parts most often covered with snow.

Near my study site, at least 15 spider species—mostly *Philodromus aureolus* and *Pityohyphantes phrygianus*—were active in spruce canopies in winter when temperatures were above freezing (Gunnarsson, 1985). In a place near Oslo at 60°N in Norway, spiders were uniformly distributed in spruce trees and occurred on almost every sampled branch from November through March. This suggests that spiders were redistributing themselves in winter (Hågvar & Hågvar, 1975, p. 25, 29). I recorded goldcrest foraging behavior only when temperatures were above 0°C both in autumn and spring, and I often saw spiders moving about, potentially offsetting any site‐specific depletion by predation. But most importantly, goldcrests frequented the outer branch parts about as often in early spring as in late autumn. Hovering did also occur in remarkably narrow spaces among twigs and needles in the interior of spruce canopies and was not restricted to the periphery (Figures 2 and 3). And again, adult goldcrests with no young to feed in June and July hovered very rarely, whereas parents feeding fledglings hovered very often, while prey distribution in spruce canopies was identical to them all (Table 6). So, site‐specific prey depletion does not seem to determine goldcrest hover frequency.

### 4.5 Spider web hindrance

Orb and sheet spider webs are common in spruce canopies in summer and early autumn but largely absent in early spring after having been swept away by snow and storms in winter. They might be a hindrance to foraging goldcrests. There is even a report of a goldcrest being entangled and stuck in a web (Tutt, 1972). The high hover frequency in autumn might be a means of avoiding spider webs. But webs usually disappear already by mid‐autumn and goldcrests, nevertheless hover‐foraged more often in November and December than in September and October (Tables 4 and 5). And while spider webs were common in June and July, goldcrests that were feeding fledglings hovered very often, whereas adults with no young to feed hovered very rarely (Table 6). The prevalence of spider webs therefore does not determine hover frequency.

## 5 CONCLUSION

None of the alternative explanations are supported. The results are highly consistent from year to year and in qualitative agreement with the working hypothesis. Notice that there is no overlap in monthly hover frequencies between autumn and spring or between providers and non‐providers in summer. The difference in hover frequency between spring and autumn is highly significant (*p* < .00004, two‐tailed randomization test), and the difference between providers and non‐providers is statistically significant (*p* = .0286, two‐tailed randomization test).

Low ambient temperatures in winter cause extra energy expenditure for thermoregulation and prolong daily foraging time. An additional aggravation is the short days with few daylight hours. Together with the high winter mortality, this suggest that goldcrests are food‐limited in winter. Food limitation eventually manifests itself as a shortage of time for foraging, which strongly promotes the use of time‐minimizing foraging methods. Hover‐foraging is favored when food is abundant in autumn but not at food scarcity in spring.

The high hover frequency of parent birds that provided for fledglings at high food density around mid‐summer probably maximized the rate of net energy gain and reproductive output. But adult non‐providers were not compelled to maximize the rate of net energy gain by using energy‐expensive hover‐foraging but were probably minimizing daily energy expenditure. Food demand obviously determined foraging effort in summer. And as food was abundant, hover‐foraging was favored by providers.

## CONFLICT OF INTEREST

None declared.

## AUTHOR CONTRIBUTIONS

**Rolf Åke Norberg:** Conceptualization (lead); data curation (lead); formal analysis (lead); funding acquisition (lead); investigation (lead); methodology (lead); project administration (lead); resources (equal); software (equal); supervision (lead); validation (lead); visualization (lead); writing‐original draft (lead); writing‐review & editing (lead).

## Data Availability

All underlying data are included within the manuscript and associated supplementary materials.
